# Do Recommendations for the Management of Hypertension Improve Cardiovascular Outcome? The Canadian Experience

**DOI:** 10.4061/2011/410754

**Published:** 2011-10-30

**Authors:** Peter Bolli, Norm R. C. Campbell

**Affiliations:** ^1^Ambulatory Internal Medicine Teaching Clinic, Department of Medicine, McMaster University, 80 King Street, Suite 2, Street Catharines (ON), Canada L2R 7G1; ^2^Departments of Medicine, Community Health Sciences and Physiology and Pharmacology, University of Calgary, Canada

## Abstract

The Canadian Hypertension Education Program (CHEP) was established in 1999 as a response to the result of a national survey that showed that a high percentage of Canadians were unaware of having hypertension with only 13% of those treated for hypertension having their blood pressure controlled. The CHEP formulates yearly recommendations based on published evidence. A repeat survey in 2006 showed that the percentage of treated hypertensive patients with the blood pressure controlled had risen to 65.7%. Over the first decade of the existence of the CHEP, the number of prescriptions for antihypertensive medications had increased by 84.4% associated with a significant greater decline in the yearly mortality from stroke, heart failure and myocardial infarction and a significant decrease in the hospitalization for stroke and heart failure. Therefore, the introduction of the CHEP and the yearly issue of updated recommendations resulted in a significant increase in the awareness, diagnosis and treatment of hypertension and in a significant reduction in stroke and cardiovascular morbidity and mortality. The CHEP model could serve as a template for its adoption to other regions or countries.

## 1. Introduction

Hypertension is still a major contributor to mortality worldwide [1], and it is estimated that there are 970 million hypertensives worldwide and it is predicted to increase to 1.56 billion in the year 2025 [2]. The risk for a fail or morbid cardiovascular or cerebral vascular event starts at the systolic blood pressure of 115 mmHg and a diastolic blood pressure of 73 mmHg. 

Therefore, it is not surprising that hypertension accounts for about 60% of strokes and 50% of heart failure [3]. Considering that lowering of systolic blood pressure by 10 mmHg and diastolic blood pressure by 5 mmHg reduces the relative risk for a coronary artery event by 23% and a stroke by 40%, it follows that blood pressure is not optimally diagnosed and treated.

There can be several reasons for this lack of diagnosing and proper control of hypertension [4]. The major factors are patient related, for example, poor adherence to treatments, physician-related inertia to properly inform the public of the danger of hypertension and the failure of physicians to diagnose, initiate, and treat blood pressure to achieve the recommended blood pressure goals.

## 2. The Canadian Situation

A Canadian national survey conducted in Canada from 1985 to 1992 revealed that 45% of individuals were unaware of their blood pressure condition, 22% were aware of having hypertension but remained untreated, 21% were treated but not controlled, and only 13% had their blood pressure treated and controlled to target, that is, less than 140/90 mmHg [5]. These results were disappointing particularly considering the easy access of Canadians to health care, but the Canadian results were similar to some European countries although better results were reported from the United States [6].

As a reaction to these results, the Canadian Hypertension Society established the Canadian Hypertension Education Program which issued the first recommendations for the management of hypertension in 1999 [7]. The mandate of the Canadian Hypertension Education Program (CHEP) was to reduce the burden of cardiovascular disease in Canada through yearly updating evidence-based recommendations for the management of hypertension, implement the recommendations, regularly evaluate and revise the program, and assess the effect of the recommendations by measuring patient outcomes. 

**Figure 1:**
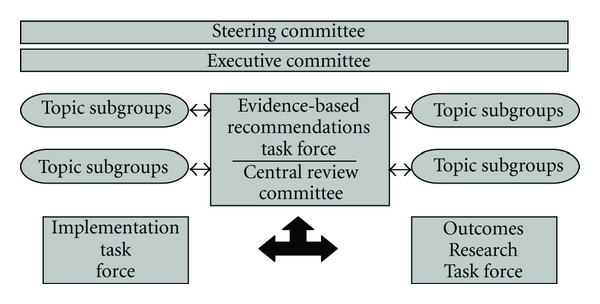
Organization of the Canadian Hypertension Education Program.

To fulfill these tasks, it required the establishment of a multidisciplinary structure as outlined in Figure 1 [8]. 

The CHEP is composed of the evidence-based task force which annually reviews and updates the recommendations according to new published information [8]. 

The implementation task force is responsible for the dissemination of the recommendations by members who have expertise in knowledge translation. Dissemination of information includes the yearly publication of the recommendations in the Canadian Journal of Cardiology which is freely available to every physician, distributing the recommendations to every practice, hospitals, public health system, the pharmaceutical industry, and local small group information meetings with physicians and nurses. Since public awareness is key to the success of improvement in hypertension management, a public education task force has developed information material for patients and the public at large through easy-to-understand pamphlets in several languages, articles in the lay press, public information meetings, and availability of a website (http://www.hypertension.ca/) [9, 10].

The Outcomes Research Task Force is responsible for evaluating and monitoring the effect of the CHEP activities on the public awareness and the management of hypertension at large. This is accomplished through a national surveillance system in collaboration with the Public Health Agency of Canada, the Canadian Institute for Health Research, the Heart and Stroke Foundation of Canada, the Canadian Stroke Network, provincial databases, and a number of other organizations [11]. The data collection includes physical measures and questionnaire surveys, morbidity and mortality data for hypertension, cardiovascular complications, and data on antihypertensive drug prescriptions. Questionnaire surveys are performed every two years to assess the prevalence of hypertension diagnosis and treatment. A national hypertension surveillance program has been initiated and has produced some initial results [11]. All this activity is overseen and directed by the executive and steering committees (Figure 1). Gradually, a number of scientific organizations, health care professionals and public health organizations became involved in the effort to improve hypertension management in Canada.

## 3. Effectiveness of the Canadian Hypertension Education Program

A national survey which was completed in 1992 showed that almost 50% of Canadians were unaware of having hypertension, 22% who were diagnosed with hypertension were not treated, and of those treated, only 13% had their blood pressure controlled below 140/90 mmHg [5]. The survey was then repeated in 2006 which showed that the proportion of Canadians who were unaware of hypertension had decreased to 16.7% and those treated and had their blood pressure controlled had risen to 65.7% [12]. This improvement was associated with a significant 84.4% increase in the number of prescriptions for antihypertensive drugs [13–15]. There was also an increased use of 2 or more antihypertensive drugs [16]. The largest increase of medications occurred in thiazide diuretics and ace inhibitors, and the smallest increase occurred in beta blockers, consistent with the CHEP recommendations not to use beta blockers as a first-line drug in patients older than 65 years of age [14, 15]. This was paralleled with an increase in hypertension-related physician office visits [14]. 

The improvement in the management of hypertension resulted in a significant reduction in cardiovascular events and stroke [13]. Since the inception of the CHEP in 1999, there was a significant greater yearly reduction in mortality due to stroke −3%, heart failure −4.3%, and −2.1% for acute myocardial infarctions. Similarly, there was also a significant reduction in the number of hospitalizations for stroke −1.6% and −3.1% for heart failure, but the rate of decline in hospitalization for acute MI remained unchanged, the reason for which is not entirely clear. The decrease in mortality correlated with the increase in the prescription for antihypertensive drugs [13]. The average yearly reductions in mortality due to heart failure, stroke, and acute MI were significantly greater than for noncardiovascular disease deaths or cancer [17]. A recent Canadian population survey confirmed the high rates of treatment and control of hypertension [18].

At the end of the first decade of the existence of the CHEP, certain factors that became important for the sustainability of the CHEP process became apparent and may serve as a guide to implement such a program in other regions or countries [19]. It needs a critical number of experts in the field to reduce individual bias and influence in order to achieve an objective result which at the end should be dictated by up-to-date evidence forming the base of the recommendations and translating data from trials and scientific studies into practical and applicable recommendations. Before issued, consensus on the content on wording of the recommendations has to be achieved. 

It also includes the participation of interdisciplinary health teams. In order to be applicable, recommendations should be revised yearly as new information becomes rapidly available. As the overseeing body, academic and government organizations are involved, which is an important factor for the integrity and credibility of the program. Although the members of the CHEP are all volunteers, certain costs have to be covered, and persistent financial support is important for the continuation of such a program.
